# Evaluating the efficacy of MEANGS for mitochondrial genome assembly of cartilaginous and ray-finned fish species

**DOI:** 10.1093/bib/bbae041

**Published:** 2024-02-12

**Authors:** Sheng-Yong Xu, Shan-Shan Cai, Zhi-Qiang Han

**Affiliations:** Fishery College, Zhejiang Ocean University, 316022 Zhoushan, China; Fishery College, Zhejiang Ocean University, 316022 Zhoushan, China; Fishery College, Zhejiang Ocean University, 316022 Zhoushan, China

**Keywords:** MEANGS, mitogenome assembly, cartilaginous fish, ray-finned fish

## Abstract

The assembly of complete and circularized mitochondrial genomes (mitogenomes) is essential for population genetics, phylogenetics and evolution studies. Recently, Song *et al*. developed a seed-free tool called MEANGS for *de novo* mitochondrial assembly from whole genome sequencing (WGS) data in animals, achieving highly accurate and intact assemblies. However, the suitability of this tool for marine fish remains unexplored. Additionally, we have concerns regarding the overlap sequences in their original results, which may impact downstream analyses. In this Letter to the Editor, the effectiveness of MEANGS in assembling mitogenomes of cartilaginous and ray-finned fish species was assessed. Moreover, we also discussed the appropriate utilization of MEANGS in mitogenome assembly, including the implementation of the data-cut function and circular detection module. Our observations indicated that with the utilization of these modules, MEANGS efficiently assembled complete and circularized mitogenomes, even when handling large WGS datasets. Therefore, we strongly recommend users employ the data-cut function and circular detection module when using MEANGS, as the former significantly reduces runtime and the latter aids in the removal of overlapped sequences for improved circularization. Furthermore, our findings suggested that approximately 2× coverage of clean WGS data was sufficient for MEANGS to assemble mitogenomes in marine fish species. Moreover, due to its seed-free nature, MEANGS can be deemed one of the most efficient software tools for assembling mitogenomes from animal WGS data, particularly in studies with limited species or genetic background information.

This journal recently published an article entitled ‘*MEANGS: an efficient seed-free tool for de novo assembling animal mitochondrial genome using whole genome NGS data*’ by Song *et al*. [1]. In their study, the authors developed a seed-free tool for *de novo* mitochondrial assembly in animals utilizing whole genome sequencing (WGS) data. MEANGS demonstrated the best overall performance compared with three other available programs, establishing itself as one of the most efficient software tools for high-quality mitochondrial genome (mitogenome) assembly [1]. This achievement is anticipated due to its recent development and incorporation of advanced bioinformatics tools designed specifically for mitogenome assembly. However, the original study reported long runtimes of up to 2 h for certain test species and identified overlap sequences in 9 out of 16 samples, with two assemblies containing multiple mitochondrial fragments rather than a single complete mitogenome sequence (refer to table 1 in [1]). These findings are noteworthy for software users and researchers focusing on mitochondrial studies, as complete and circularized mitogenomes are critical for downstream population genetics, phylogenetics and evolutionary investigations. Overlapped or missing regions in the assembly results require manual editing and correction, which is time-consuming and inconvenient. Similarly, in our recent studies involving the assembly of mitogenomes for marine fish species such as the white-spotted bamboo shark (*Chiloscyllium plagiosum*), ocellate spot skate (*Okamejei kenojei*) and brown-spotted flathead (*Platycephalus* sp.1) using MEANGS in both default and deepin modes, we also detected overlap sequences. These identified shortcomings raise concerns among users regarding the performance of MEANGS in mitogenome assembly.

A thorough understanding of a software’s functionalities and utilizing them to their fullest potential is crucial for effective utilization. In the case of MEANGS, the authors briefly mentioned two useful function modules, namely the data-cut function and circular detection module, in the discussion section of their original article [1]. The data-cut function significantly reduces runtime, while the circular detection module detects potential clips and removes overlapped sequences to facilitate assembly circularization. As a result, the original work achieved high efficiency with some test samples, completed in less than 10 min (refer to table 1in [1]). In this study, we evaluated the effectiveness of MEANGS in assembling mitogenomes of various cartilaginous and ray-finned fish species. Our assessments revealed the excellent performance of MEANGS in most species when employing the data-cut function (parameter: -n) and circular detection module (parameter: –clip) in both default and deepin modes (Table 1). The data-cut function enables subsampling of input WGS data, substantially reducing runtime and memory usage [1]. MEANGS successfully assembled complete mitogenome sequences from the WGS data of seven species, even with data sizes of up to 280 Gb, achieving a runtime of approximately 3 min. However, the assembly times for the pufferfish *Takifugu rubripes*, sterlet *Acipenser ruthenus* and tarpon *Megalops atlanticus* were longer, taking around 20, 90 and 140 min, respectively. The data for these three species were obtained from the National Center for Biotechnology Information (NCBI) database, while the others were our own collected data. These differences in runtime may be attributed to the influence of raw data from online databases. In their original article [1], the authors proposed that ~30× coverage of the mitogenome was sufficient for assembly. However, estimating mitogenome coverage directly from WGS data is challenging. In this study, we utilized the data-cut function to determine the minimum amount of WGS data required for MEANGS analysis. The results indicated that subsampled reads ranging from 400 000 to 700 000 were sufficient for complete mitogenome assembly in seven investigated species (Table 1), suggesting that approximately 0.2 Gb of WGS data are adequate, with a runtime of approximately 2 min. Inconsistently, the minimum WGS data required for *T. rubripes* were approximately 0.6 Gb, and the runtime was around 11 min. On the other hand, *A. ruthenus* and *M. atlanticus* required minimum WGS data of about 8.0 and 13.5 Gb, respectively, with a runtime exceeding 1 h. Given the comparatively smaller size of *T. rubripes*’ genome (approximately 0.3 Gb [2]) among marine fish species, it is suggested that WGS clean data with coverage of about 2× might suffice for MEANGS to assemble mitogenomes of marine fish species. Inconsistently, the sterlet *A. ruthenus* had a sequencing coverage of around 4×, based on its genome size of 1.8 Gb [3]. The tarpon *M. atlanticus*, with a genome size of 1 Gb [4], had an estimated sequencing coverage of around 15×. To further validate this conclusion, additional samples and different tissue types (e.g. muscle, blood, etc.) should be tested to avoid any influence from tissue-specific mitochondrial DNA content [5]. In addition, it is important to note that using the minimum data can lead to inconsistent assembly results, likely due to the randomness of subsampled data. As the input WGS data increase, the assembly results will become more consistent.

**Table 1:** Performance on 10 fish species of MEANGS for mitogenome assembly

Species	Input data (Gb)	MEANGS				Reference
		Parameter setting	Runtime	Assembly length (bp)	Integrity and accuracy^*^	
*Chiloscyllium plagiosum*	282	-n 1000 000	3min01s	28 512	100%; 100%	NC_012570.1 (16 726 bp)
		-n 1000 000–clip	3min01s	16 726	100%; 100%	
		-n 500 000–clip	1min49s	16 726	100%; 100%	
		-n 400 000–clip	1min18s	13 151^a^	78.63%; 100%	
*Okamejei kenojei*	61	-n 1000 000	3min07s	28 857	100%; 100%	NC_007173.1 (16 972 bp)
		-n 1000 000–clip	3min07s	16 973	100%; 99.99%	
		-n 700 000–clip	2min18s	16 973	100%; 99.99%	
		-n 600 000–clip	2min02s	16 984^b^	100%; 99.93%	
*Scoliodon macrorhynchos*	26	-n 1000 000	3min07s	28 644	100%; 100%	NC_018052.1 (16 693 bp)
		-n 1000 000–clip	3min07s	16 693	100%; 100%	
		-n 500 000–clip	1min52s	16 693	100%; 100%	
		-n 400 000–clip	1min27s	16 692^b^	100%; 99.99%	
*Sebastes schlegelii*	38	-n 1000 000	2min55s	16 931	100%; 100%	NC_005450.1 (16 525 bp)
		-n 1000 000–clip	2min55s	16 779	100%; 98.46%	
		-n 600 000–clip	2min06s	16 779	100%; 98.46%	
		-n 500 000–clip	1min51s	16 801^b^	100%; 98.33%	
*Lophius litulon*	61	-n 1000 000	3min07s	18 783	100%; 100%	NC_023828.1 (16 431 bp)
		-n 1000 000–clip	3min07s	16 468	100%; 99.77%	
		-n 600 000–clip	2min03s	16 468	100%; 99.77%	
		-n 500 000–clip	1min54s	16 486^b^	100%; 99.66%	
*Engraulis japonicus*	46	-n 1000 000	3min09s	28 527	100%; 100%	NC_003097.1 (16 675 bp)
		-n 1000 000–clip	3min09s	16 675	100%; 100%	
		-n 400 000–clip	1min41s	16 675	100%; 100%	
		-n 300 000–clip	1min20s	16 678^b^	100%; 99.98%	
*Platycephalus* sp.1	84	-n 1000 000	3min06s	16 692	100%; 100%	MT628402.1 (16 552 bp)
		-n 1000 000–clip	3min06s	16 552	100%; 100%	
		-n 700 000–clip	2min19s	16 552	100%; 100%	
		-n 600 000–clip	2min00s	16 537^b^	100%; 99.91%	
*Takifugu rubripes*	7	-n 3000 000	19min53s	16 559	100%; 100%	NC_004299.1 (16 447 bp)
		-n 3000 000–clip	19min53s	16 448	100%; 99.99%	
		-n 2000 000–clip	10min42s	16 448	100%; 99.99%	
		-n 1900 000–clip	10min11s	16 707	100%; 98.42%	
*Acipenser ruthenus*	114	-n 30 000 000	92min13s	28 956	100%; 100%	LR824036.1 (16 706 bp)
		-n 30 000 000–clip	92min13s	16 705	100%; 99.99%	
		-n 27 000 000–clip	75min46s	16 705	100%; 99.99%	
		-n 25 000 000–clip	72min33s	16 857^b^	100%; 99.10%	
*Megalops atlanticus*	80	-n 50 000 000	144min01s	16 686	100%; 100%	NC_005804.1 (16 686 bp)
		-n 50 000 000–clip	144min01s	16 686^b^	100%; 100%	
		-n 45 000 000–clip	135min03s	16 686^b^	100%; 100%	
		-n 40 000 000–clip	97min11s	16 890^b^	100%; 98.78%	

Note: ^a^The output file contained a total of four contigs, with lengths of 1303, 2106, 3842 and 5900 bp, respectively. ^b^The output file contained one contig, yet no candidate clip point is found using the circular detection module. ^*^The integrity and accuracy of an assembly result were evaluated following the description in [1].

The circular detection module within MEANGS is critical in identifying candidate clips and eliminating overlapping sequences from the assembly results [1]. This functionality is vital for achieving effective assembly circularization. When this module was not utilized, redundant and overlapping fragments were detected in the assembly results (Table 1). Conversely, using the ‘–clip’ parameter, the majority of our assembly results were either identical or differed by just one base pair from the reference sequences of the corresponding species, making them suitable for downstream annotation and genetic analyses. It is worth noting that the MEANGS assembly results for the rockfish *Sebastes schlegelii* and the anglerfish *Lophius litulon* evidently differed from the reference sequences (Table 1). Further alignment analysis revealed the presence of two regions (279 and 40 bp fragments) in the control region that were not aligned in the assemblies of *S. schlegelii* and *L. litulon*, respectively. Unexpectedly, the blast results against the GenBank database indicated that these two unmapped sequences matched with other *Sebastes* spp. (*Sebastes aleutianus*, with the highest identity of 87%) and *Lophius piscatorius* (with an identity of 98%) (Supplementary data). Additional programs, such as NOVOPlasty [6], were utilized to assemble the mitogenomes of these two species. The assembled results were found to be identical to those obtained through MEANGS, indicating the accuracy of our assemblies. Still, further confirmation of the assembly results for *S. schlegelii* and *L. litulon* is necessary through polymerase chain reaction amplification and Sanger sequencing. Despite this, we firmly believe that the data-cut function and circular detection module are vital safeguards that contribute to the exceptional performance of MEANGS. Therefore, we highly recommend that users employ the ‘-n’ and ‘–clip’ parameters when utilizing MEANGS for mitogenome assembly.

In conclusion, MEANGS demonstrates outstanding performance in the assembly of mitogenomes for the investigated fish species, even when handling large WGS datasets. Apart from its notable assembly completeness and efficiency, MEANGS possesses the advantage of being seed-free. By eliminating the need for manual seed retrieval, selection and input [1], MEANGS enables the automatic assembly of mitogenomes, making it particularly valuable for studies involving species that lack relevant taxonomic and genetic background information [7]. This feature greatly facilitates research on ancient DNA, environmental DNA and forensic DNA. Furthermore, considering the efficacy of MEANGS in extracting mitogenomes from various animal categories, including Arthropoda, Bryozoa, Chordata, Echinodermata, Mollusca and Nematoda (refer to the species_class in the readme file of MEANGS for detailed information), we firmly believe that MEANGS will greatly enhance investigations into the phylogeny, evolution and genetics of animals utilizing mitogenomic approaches.

**Key Points**

- The data-cut function embedded in MEANGS can subsample input WGS data to largely increase efficiency, especially for large WGS datasets.
- The circular detection module in MEANGS can detect candidate clips and cut overlap sequences, which is crucial for the assembly circularization.
- We strongly recommend users employ the ‘-n’ and ‘–clip’ parameters when using MEANGS for mitogenome assembly.

## Data Availability

The whole genome sequencing data have been deposited in the NCBI Sequence Read Archive (SRA) database under accession numbers PRJNA823509 (*C. plagiosum*), PRJNA1004886 (*O. kenojei*), PRJNA507287 (*S. schlegelii*), PRJNA640729 (*Platycephalus* sp.1) and PRJNA1034949 (*L. litulon*, *Engraulis japonicas*, *Scoliodon macrorhynchos*). The test data of pufferfish *T. rubripes* (SRR12009751), sterlet *A. ruthenus* (ERR6363242) and tarpon *M. atlanticus* (SRR17844086) were downloaded from the NCBI SRA database. The related outputs of MEANGS and supplementary data have been deposited in the Figshare repository (https://doi.org/10.6084/m9.figshare.23921238.v5).

## Author Biographies

**Sheng-Yong Xu** is an associate professor at the Fishery College, Zhejiang Ocean University. His research interests include genomics, bioinformatics and population genomics.

**Shan-Shan Cai** is a lab master at the Fishery College, Zhejiang Ocean University. Her research interests include mitogenomics and population genetics.

**Zhi-Qiang Han** is a PhD professor at the Fishery College, Zhejiang Ocean University. His research interests include genomics, evolutionary biology and ecological genomics.
